# Fever after Vaccination against SARS-CoV-2 with mRNA-Based Vaccine Associated with Higher Antibody Levels during 6 Months Follow-Up

**DOI:** 10.3390/vaccines10030447

**Published:** 2022-03-14

**Authors:** Andrea Kanizsai, Tihamer Molnar, Reka Varnai, Laszlo Zavori, Margit Tőkés-Füzesi, Zoltan Szalai, Janos Berecz, Peter Csecsei

**Affiliations:** 1 Department of Dentistry, Medical School, University of Pecs, 7624 Pecs, Hungary; kanizsai.andrea@pte.hu; 2 Department of Anaesthesiology and Intensive Care, Medical School, University of Pecs, 7624 Pecs, Hungary; molnar.tihamer@pte.hu; 3 Department of Primary Health Care, Medical School, University of Pecs, 7624 Pecs, Hungary; varnai.reka@pte.hu; 4 Salisbury NHS Foundation Trust, Salisbury SP2 8BJ, UK; laszlo.zavori@nhs.net; 5 Department of Laboratory Medicine, Szigetvár Hospital, 7900 Szigetvar, Hungary; 6 Department of Internal Medicine, Szigetvár Hospital, 7900 Szigetvar, Hungary; drszalzo@gmail.com; 7 Szigetvár Hospital, 7900 Szigetvar, Hungary; berecz.janos01@gmail.com; 8 Department of Neurosurgery, Medical School, University of Pecs, 7624 Pecs, Hungary; csecsei.peter@pte.hu

**Keywords:** COVID-19, anti-SARS-CoV-2, spike IgG, mRNA vaccine, adverse reaction, fever

## Abstract

Background: The effect of post-vaccination adverse events on immunogenicity is unknown. We aimed to explore relationship between post-vaccination adverse reactions and antibody levels during 6-month follow-up. Methods: Blood was serially drawn from healthcare workers after the second dose of BNT162b2 mRNA vaccine (Day 12, 30, 60, 90, 120, 150, and 180) and anti-SARS-CoV-2 spike IgG (S-IgG) levels were measured. Following each vaccine dose, volunteers completed a questionnaire regarding adverse reactions (symptomatic vs. asymptomatic groups). Results: A total of 395 subjects received the second dose of the vaccine. The main results were as follows: (i) fever after the 2nd dose was independently associated with the median S-IgG level at all follow-up time points; (ii) significantly higher S-IgG levels were observed in the symptomatic group of patients without prior COVID-19 infection throughout the entire follow-up period; (iii) prior COVID-19 positivity resulted in higher S-IgG levels only in the asymptomatic group from Day 90 of the follow-up period; (iv) both prior COVID-19 disease with asymptomatic status and symptomatic status without prior COVID-19 infection resulted in similar S-IgG antibody levels; (v) significantly lower serum S-IgG levels were observed in smokers. Conclusion: Fever may play an important role in the post-vaccination immune response in the long term.

## 1. Introduction

According to the WHO database [1], more than 390 million confirmed SARS-CoV-2 infections worldwide since 2019 with more than 5.5 million deaths. Beside the devastating health effects, the novel coronavirus disease 2019 (COVID-19) also had harmful economic and social consequences. Controlling the pandemic required joint and rapid action by science and pharmaceutical companies leading to the development of perhaps the most important vaccines in human history: the mRNA-based vaccines against SARS-CoV-2. A two-dose regimen of BNT162b2 and mRNA-1273 were found to be safe and more than 90% effective against COVID-19 [2,3]. However, several systemic adverse reactions (AR) were observed during vaccination, mainly after the second dose. The most common ARs were fatigue (59–65%), headache (52–58%), fever (16%), and chills (44%) [2,3]. The rapid pace of vaccine development and the uncertainty of potential long-term adverse effects raised some level of hesitation against mRNA vaccines in the global community [4]. By 21 January 2022, 60.3% of the world’s population had received at least one COVID-19 vaccine, making it the world’s largest vaccination campaign ever [5]. However, potential relationship between vaccine-related ARs and immunogenicity has been strongly disputed, poorly investigated and the currently available evidence is contradictory [6,7]. The main objective of the present study was to elucidate the long-term effect of such ARs on antibody production in healthcare workers with and without prior COVID-19 infection.

## 2. Materials and Methods

### 2.1. Study Design and Population

From 27 January 2021 to 9 May 2021, health care workers in Szigetvar Hospital were recruited for the present study. Participants were scheduled to initiate BNT162b2 mRNA (Pfizer/BioNTech, Comirnaty, Reinbek, Germany) vaccination according to the original protocol of 2 doses with a 3-week interval. Venous blood samples were collected at seven time points, namely 12 and 30, 60, 90, 120, 150, and 180 days following the second vaccine dose (designated Day 12, Day 30, Day 60, Day 90, Day 120, Day 150 and Day 180, respectively). Participants with evidence of COVID-19 infection were also included; COVID-19 had to be diagnosed 3–5 months prior to the study by RT-PCR (reverse transcription-polymerase chain reaction). Before administration of the 1st dose, history of hypertension, diabetes, hypothyreosis, autoimmune disease, malignancies, smoking, recent flu vaccination and allergies were recorded and an inquiry into age, sex, height, body weight, use of medications, including non-steroid anti-inflammatory drugs (NSAIDS), statins, antihypertensives, ACE inhibitors, beta blockers, calcium channel blockers, immunosupressants, statins, platelet inhibitors, steroids was performed using a questionnaire. Based on the presence of vaccination induced adverse reactions; (i) symptomatic (adverse reactions within 7 days after each dose) vs. asymptomatic (no adverse reaction occurred after any dose), and (ii) prior COVID-19 infection status, the following subgroups were created: prior COVID-19 negative and asymptomatic individuals (Group 1); prior COVID-19 negative and symptomatic individuals (Group 2); prior COVID-19 positive, but asymptomatic patients (Group 3) and prior COVID-19 positive and symptomatic patients (Group 4).

### 2.2. Adverse Reaction Assessment

ARs after the 1st vaccination were recorded immediately before the administration of the 2nd dose and adverse reactions after 2nd dose were recorded on Day 12 follow-up visit along with first sampling. ARs were investigated in a questionnaire where the volunteer was required to clearly indicate if they experienced an adverse reaction within 1 week after vaccination. Volunteers had to select the symptoms they experienced within 1 week after vaccination from the following list: local pain, fatigue, fever, myalgia, arthralgia, headache, chills, nausea, lymph node swelling, or other (free description).

### 2.3. Measurement of Antibody Titers

Blood samples for measurements were drawn from volunteers via veinipuncture with a 21-gauge needle into a closed system blood sampling serum separator tube without anticoagulant (Vacuette^®^, Greiner Hungary LTD, Mosonmagyaróvár, Hungary). Samples were tested for IgG antibodies against SARS-CoV-2 spike proteins in peripheral blood on a fully automated benchtop Access2 analyzer (Beckman Coulter Hungary LTD, Budapest, Hungary) according to the manufacturer’s instructions. For the determination of antibodies against the SARS-CoV-2 spike protein we used the Beckman-Coulter Access SARS-CoV-2 IgG II assay (Beckman Coulter Hungary LTD). The test measures IgG antibodies directed to the receptor-binding domain (RBD) of the spike protein of the coronavirus. The two-step enzyme assay is a paramagnetic particle, chemiluminescent immunoassay intended for the semi-quantitative determination of IgG antibodies against SARS-CoV-2 in human serum. Briefly, a sample is added to a reaction vessel with buffer and paramagnetic particles coated with recombinant SARS-CoV-2 protein. After incubation in a reaction vessel, materials bound to the solid phase are held in a magnetic field while unbound materials are washed away. A monoclonal anti-human IgG alkaline phosphatase conjugate is added to the mix and the conjugate binds to the IgG antibodies captured on the particles. A second separation and wash step removes any unbound conjugates. A chemiluminescent substrate is added to the vessel and the light generated by the reaction is measured with a luminometer. The light-production is directly proportional to the concentration of SARS-CoV-2 IgG antibody in the sample. The amount of the antibodies in the sample is determined from a multi-point calibration curve. The results were interpreted as follows: cut-off index < 10 AU/mL as non-reactive and reactive ≥ 10 AU/mL.

### 2.4. Ethics Statement

This study is covered by approval from the Hungarian National Public Health Centre (40576-8/2021/EÜIG). All procedures were performed in accordance with the ethical guidelines of the 1975 Declaration of Helsinki. Written informed consent was provided by all participants before enrollment in the present study.

### 2.5. Statistical Analysis

Summary statistics of the participants were constructed using frequencies and proportions for categorical data, and mean and standard deviation (SD) for continuous variables. Statistical analysis was performed using SPSS version 23.0 (IBM Corporation, Armonk, NY, USA). Conformity of data to normal distribution was determined by histogram and Kolmogorov–Smirnov test. The between-group difference was calculated with χ2, Fisher’s exact, Mann–Whitney U, and Kruskal–Wallis tests in line with suitability. The significance level was considered as *p* < 0.05. Data with nonparametric distribution were presented as median and interquartile range (IQR). Correlations of Ig levels with adverse reactions were tested by linear regression using Spearman correlation coefficient (R).

## 3. Results

### 3.1. Study Participants

Between 10 February and 13 June 2021, a total of 395 people received the second dose of Pfizer-BioNTech vaccine (BNT162b2) and provided informed consent for study enrollment. From these, 383 individuals completed the questionnaire on post-vaccination ARs and gave post-vaccination blood samples at Day 12, 323 at Day 30, 320 at Day 60, 303 at Day 90, 268 at Day 120, 220 at Day 150, and 279 at Day 180. The age of the vaccinated volunteers ranged from 20 to 77 years (median 47 years; IQR 39–55). 76.7% were females and 34.7% were current smokers. A total of 169 (44.1%) subjects had at least one AR within 7 days of any vaccination (symptomatic group), and 214 (55.9%) reported no vaccine-related ARs (asymptomatic group). There were significantly more patients with history of allergy in the symptomatic group. The characteristics of the participants are shown in Table 1.

### 3.2. Adverse Reactions

ARs occurred in 125 patients after the first dose and in 131 after the second dose. The total number of ARs within 7 days after the first vaccination was 314, while 365 ARs occurred within 7 days after the second dose. In 87 participants (22.7%) at least one AR occurred after both vaccinations and in 214 cases (55.9%) no ARs occurred after either dose. The most common ARs during vaccinations were myalgia (27.8%) and local pain (19.7%). A detailed description of adverse reactions is shown in Table S1.

### 3.3. Relationship between Antibody Levels, Demographics, and Clinical Variables

**Figure 1:**
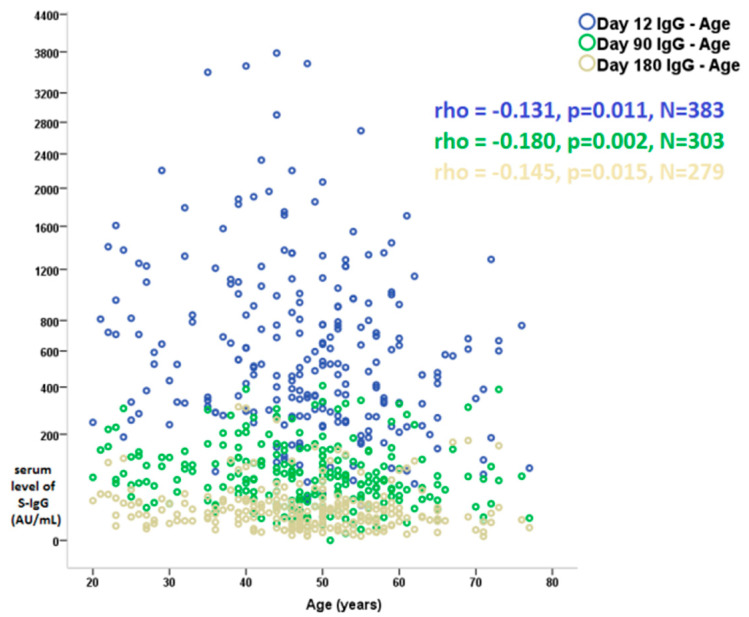
Correlation of serum level of S-IgG and age at Day 12, Day 90, and Day 180 follow-up visit after the 2nd dose of BNT162b2 mRNA (Pfizer/BioNTech, Comirnaty) vaccine. Values are Spearman correlation coefficients (rho). S-IgG; anti-spike immunoglobulin G, mRNA; messenger ribonucleic acid.

Age showed a negative correlation with serum antibody levels at all time points in this follow-up study (Figure 1; data of Day 30, 60, 120, and 150 are not displayed). Significantly lower serum S-IgG antibody levels were observed in smoking individuals over the entire 6-month study period when compared to non-smokers (Table S3). Neither female gender nor BMI showed a significant association with antibody production during follow-up. A mild negative correlation was observed between antibody production and ACE inhibitor and statin use respectively, while oral contraceptive treatment was associated with higher antibody levels in the first month.

### 3.4. Relationship between Antibody Levels and Adverse Reactions

**Figure 2:**
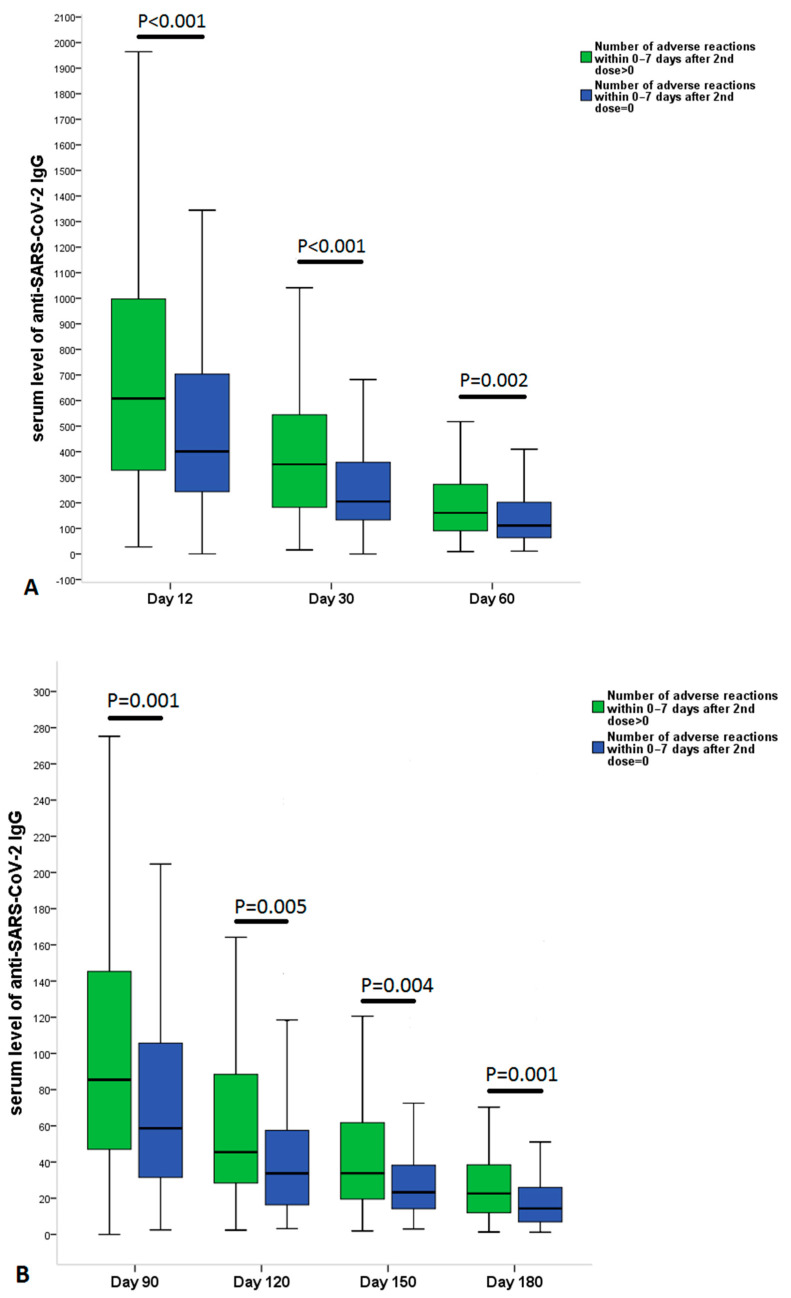
Comparison of serum level of anti-SARS-CoV-2 IgG at (**A**) 12, 30, 60 and (**B**) 90, 120, 150, 180 days after the 2nd dose of vaccination (BNT162b2 mRNA) in patients without or with at least one adverse reaction after each vaccine dose. The data are provided as median and interquartile range. The between-group differences were calculated by the Kruskal–Wallis test.

**Figure 3:**
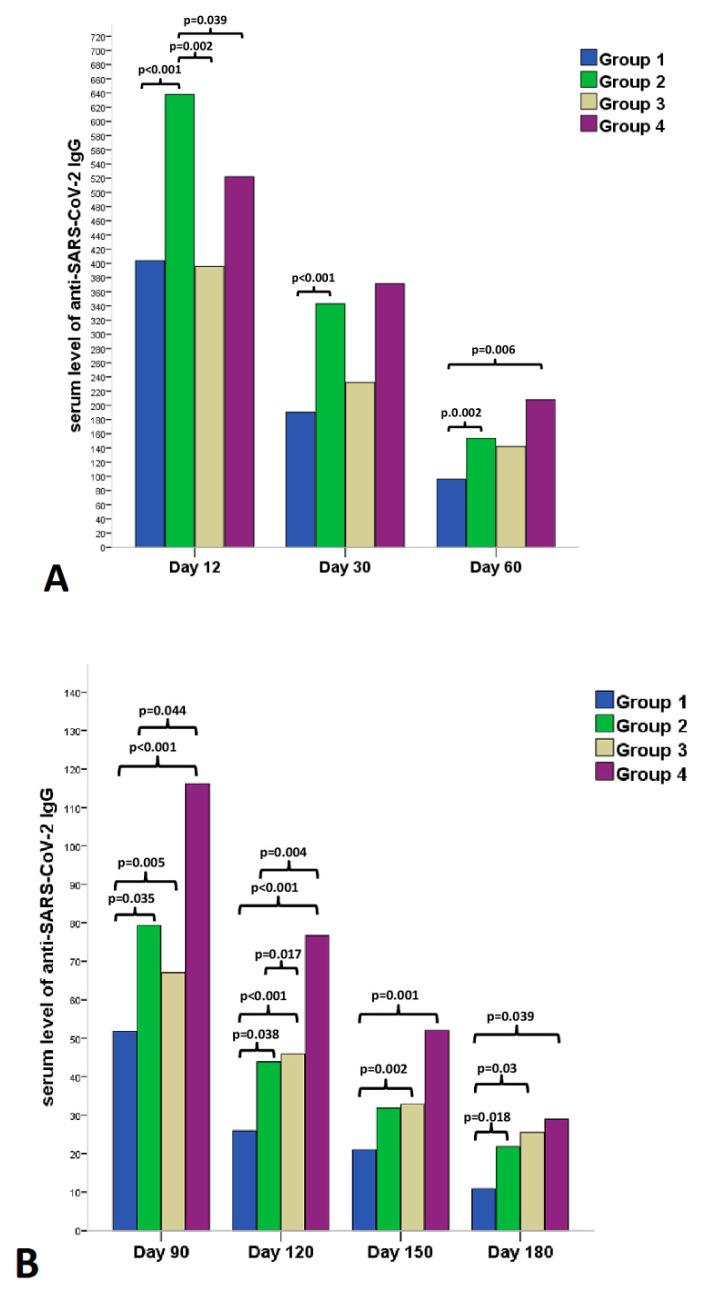
Comparison of serum levels of anti-SARS-CoV-2 IgG at (**A**) 12, 30, 60 and (**B**) 90, 120, 150, 180 days after the 2nd dose of vaccination (BNT162b2 mRNA). Healthcare workers were divided into four study groups: Group 1 = individuals without prior SARS-CoV-2 infection and with no adverse reaction after vaccination; Group 2 = individuals without prior SARS-CoV-2 infection and with at least one adverse reaction after vaccination; Group 3 = individuals with prior SARS-CoV-2 infection and with no adverse reaction after vaccination; Group 4 = those who had prior SARS-CoV-2 infection and at least one adverse reaction after vaccination. Sample size at each follow-up time point is shown in table below.




After the 1st dose fever, chills, and muscle pain showed a strong positive correlation with antibody levels during the 6-month follow-up period. However, after the 2nd dose the strongest positive correlation with antibody titer was observed for fever and chills (Table S2). Significantly higher serum anti-SARS-CoV-2 spike IgG antibody levels were observed at all time points of the six-month follow-up period in the symptomatic group (Figure 2A,B). After grouping patients according to previous COVID-19 infection and adverse reactions after vaccinations, the following results were observed in antibody levels (Figure 3A,B): (i) At the earliest time point at follow-up (Day 12) symptomatic COVID-19 negative patients (Group 2) had the highest antibody levels among the groups; (ii) COVID-19 negative and symptomatic patients (Group 2) had higher antibody levels during the entire 6-month follow-up period than COVID-19 negative and asymptomatic patients (Group 1) (Figure 3A,B); (iii) in the first 60 days (Day 12, Day 30 and Day 60) COVID-19 positive status has not led to significantly higher antibody levels in the asymptomatic group compared to COVID-19 negative individuals. This trend was reversed from Day 90, because prior COVID-19 positivity resulted in significantly higher antibody levels at 90-, 120-, 150-, and 180-day follow-up visits in the asymptomatic group. Interestingly, COVID-19 positive but asymptomatic subjects (Group 3) and COVID-19 negative but symptomatic individuals (Group 2) produced similar antibody levels over the 6-month follow-up period, except initial levels at Day 12. 

**Day 12**

**Day 30**

**Day 60**

**Day 90**

**Day 120**

**Day 150**

**Day 180**
Group 116712913612910890110Group 213111911511510984107Group 347423647262127Group 438333328242425Data are presented as medians and IQR.


A statistical analysis was run at all follow-up time point with median value of S-IgG as the outcome of interest. Based on binary logistic regression analysis, fever after 2nd dose proved to be an independent predictor of median S-IgG level at all follow-up time points (Table 2, Table S4).

## 4. Discussion

In this prospective, single-center follow-up study serum anti-SARS-CoV-2 spike Ig antibody levels were serially recorded in healthcare workers at 12, 30, 60, 90, 120, 150, and 180 days after the 2nd dose of BNT162b2 vaccine. The key results of this study are the following: (i) On day 12 after administration of the 2nd dose, volunteers with at least one vaccine related adverse reaction (symptomatic group) had the highest S-IgG antibody levels, regardless of prior COVID-19 status; (ii) significantly higher S-IgG levels were observed in the symptomatic group of subjects without prior COVID-19 infection when compared to the asymptomatic group throughout the entire follow-up period; (iii) in the asymptomatic groups prior COVID-19 positivity (Group 3) resulted in higher S-IgG levels from only Day 90 of the follow-up period compared to Group 1; (iv) prior COVID-19 disease with asymptomatic status (Group 3) and symptomatic status without prior COVID-19 (Group 2) infection resulted in nearly identical, not significantly different S-IgG antibody levels; (v) fever after the 2nd dose was independently associated with higher median S-IgG level at all follow-up time points.

In our study, we observed significantly lower serum S-IgG antibody titers in older individuals, which is consistent with the results previously reported in the literature [7,8]. A previous study demonstrating that aging decreased antibody response among COVID-19 patients and the fact that aged people demonstrated weaker immunologic responses [7]. 

Coordination of SARS-CoV-2 antigen-specific responses was disrupted in individuals ≥ 65 years old, resulting in an uncontrolled response between CD4 + and CD8 + cells and antibody production that can lead to failure of disease control [8]. Significantly lower serum antibody levels were observed in smoking subjects over the entire 6-month study period when compared to non-smokers. In addition, smoking status was an independent predictor of the median S-IgG level at Day 60, 120, and 180 follow-up visits. However, we have no information on the proportion of seroconversion among smoking and non-smoking volunteers. There is more evidence that smoking lowers serum IgG levels. Smoking was associated with a decrease in serum IgG levels in a small case-control study [9]. In a larger study of 1787 patients, it was found that cigarette smoking was associated with reduced IgG median concentrations [10]. There are several explanations for the effect of smoking on the humoral immune response. These might include direct effects on B cells and indirect effects on T cells and antigen-presenting cells, which could affect Ig class switching and/or differential survival of naive B cells or memory B cells [11]. Activity of nicotinic acetylcholine receptors can suppress B-cell activation in response to antigenic challenge [12]. In smokers, we observed significant negative correlation with antibody response to vaccination for a minimum of six months, suggesting that smoking affects the immunogenicity of vaccines in our cohort.

In this study we did not observe a significant difference between genders in terms of antibody response, however, the majority of participants were female. Several mechanisms can cause a different antibody response between males and females such as hormonal, genetic, and microbiota differences [13]. Growing body of data provide evidence that sex-specific effects may lead to different outcomes of vaccine safety and efficacy [14]. Therefore, it would be important that sex-based differences were to be considered and investigated in pre-clinical and clinical trials.

In our study, systemic events such as chills and fever showed a strong correlation with subsequent antibody response against SARS-CoV-2 spike protein. Besides, fever after the 2nd dose proved to be an independent predictor of median S-IgG level at all follow-up time points. Naaber et al. found that fever was significantly associated with the spike-receptor binding domain (S-RBD) IgG levels at 1, 6, and 12 weeks after second dose of COVID-19 mRNA Comirnaty (Pfizer-BioNTech) vaccine [15]. The importance of body temperature elevation in an adequate immune response was previously highlighted. Physiological temperature change like fever acts to regulate the emergence of new immune responses but does not restrict the activity of existing effector mechanisms once they have been formed [16]. There is a growing body of evidence suggesting that febrile temperatures boost the effectiveness of the immune response during infections by stimulating both the innate and adaptive arms of the immune system [17]. These previous evidence and our results both confirm that the attenuation and elimination of fever in any form (such as the use of NSAIDs) at the beginning of the immune response may adversely affect the immune process, even in the long run.

Coggins et al. found no correlation between symptom severity following the first or second vaccine doses and IgG reactivity with spike protein, but at the same time a significant correlation was observed with duration of symptoms after the second shot of vaccination and anti-spike IgG titers (1). Müller et al. found that there was not any general correlation between vaccination-induced SARS-CoV-2 spike-specific IgG or neutralizing antibody production and the presence or absence of individual post-vaccination reaction reports [18]. In contrast, a study with the H1N1 vaccine found that titers were 60% higher in children with fever ≥ 38 °C after vaccination, suggesting an enhanced immune response in those who had side effects after vaccination [19]. During the examination of hospital workers who received a prime-boost vaccination with BNT162b2, only a weak but existing correlation was found between the ARs and SARS-CoV-2 antibody levels [20]. In contrast, Hwang et al. concluded after vaccination of 135 healthy individuals with either AZD1222 (AstraZeneca) or BNT162b2 (Pfizer/BioNTech) that the local and systemic reactogenicity may not be associated with humoral immunogenicity [21]. However, in two recent studies a clear correlation was found between systemic adverse events including fever and antibody titer following COVID-19 vaccination, which is also consistent with our results [7,22]. The literature on the relationship between reactogenicity and immunogenicity of vaccines is limited and contradictory. The inconsistent results shown in the studies are difficult to explain. One possible explanation is that there is no information about the medications taken before and after vaccination, especially regarding the use of NSAIDs. Two (consecutive, randomized controlled, open-label) vaccination studies provided evidence that after vaccination of infants with a ten-valent pneumococcal non-typeable Haemophilus influenzae protein D-conjugate vaccine (PHiD-CV) co-administered with the hexavalent diphtheria-tetanus-3- component acellular pertussis-hepatitis B-inactivated poliovirus types 1, 2, and 3-H influenzae type b (DTPa-HBVIPV/Hib) and oral human rotavirus vaccines, antibody concentration was significantly lower in the group receiving prophylactic paracetamol than in the group not receiving it [23]. Thus, it is hypothesized that the use of regular or occasional analgesic NSAIDs (e.g., paracetamol) in the peri-vaccination period may affect the production of antibodies. This assumption is supported by several previous evidence. Bancos et al. reported that a panel of widely used NSAIDs blunts antibody synthesis in human peripheral blood mononuclear cells and in purified B cells [24], and it reduces antibody synthesis which may negatively affect the post-vaccination immune response. NSAIDs suppress T-cell activation by inhibiting p38 MAPK induction, thus the immunosuppressant activity of NSAID on T-cells underlines the role of COX activity in the normal process of lymphocyte activation [25]. Ryan at al. found evidence that NSAIDs and the new Cox-2-selective drugs negatively affect B-cell function and attenuate antibody production in humans [26]. NSAIDs are one of the most commonly used drugs, are recommended for all age categories, and are prescribed for relieving transient pain, therefore, their uncontrolled use might affect post-vaccination side effects and may alter the humoral immune response to antigen stimuli. However, the mechanism by which antipyretic analgesics reduce antibody response remains unclear and not fully explained by COX enzyme inhibition, and the involvement of nuclear and subcellular signaling pathways also arises [27]. More detailed immunological studies are needed to accurately determine the effect of NSAIDs or other antipyretic or analgesic drugs on the vaccine-induced immune response.

Overall, these evidence may explain the contradictory results in the literature between post-vaccination adverse effects and antibody production. 

Morales et al. provided evidence that a single dose of the BNT162b2 vaccine could be sufficient to confer a similar immunization in those patients with previous history of COVID-19 (individuals vaccinated at least 3–5 months after SARS-CoV-2 infection) [28]. This hypothesis was supported by others’ work [29]. In a cohort of 1025 individuals, a steeper slope of decline for IgG and neutralizing antibodies was found in vaccinated individuals without previous COVID-19 infection compared to those with previous COVID-19 infection [30]. IgG antibodies in most patients with COVID-19 can last for at least 12 months after discharge and the IgG titers decreased significantly in the first 6 months and remained stable in the following 6 months [31]. These results support the findings of our study that previous COVID-19 infection compensates for the decrease in antibody levels following vaccination, an effect that occurs primarily in the late phase beyond 90 days. In a recent systematic review and meta-analysis, significantly more ARs were reported in vaccine groups compared with placebo groups after COVID-19 vaccination trials, but the rates of reported ARs in the placebo arms were still substantial [32]. The result of this work is remarkable, however, the study did not examine the rate of seroconversion or subsequent antibody response in the placebo group and the vaccine group. In addition, the correlations observed in our study (correlation between post-vaccination fever and antibody titer) showed a robust association for 6 months.

The strength of our results is the relatively large number of volunteers and the long follow-up period. A significant limitation of our study is that the frequency of NSAID and paracetamol use after vaccination was not recorded in our study questionnaire. Thus, their potential effect on the association between antibody production and adverse events after vaccination cannot be established. Furthermore, the male population was underrepresented and conclusions on gender differences in vaccine response is limited. Some volunteers missed follow-up dates, which reduced the number of participants at consecutive follow-up times, but the statistical power of our results remained strong. To accurately report adverse reactions, volunteers kept a diary. Using this will help reduce the possibility of recall bias, but it cannot be completely ruled out.

## 5. Conclusions

Several factors have an impact on antibody levels after SARS-CoV-2 vaccination including age, smoking status, prior COVID-19 positivity, and adverse reactions after each dose of vaccines. Fever was associated with higher median S-IgG level during a 6-month follow-up period. These results may convince those who refuse vaccination due to fear of vaccination reactions. In addition, an individual approach that takes all factors influencing antibody levels into account might be useful when developing a vaccination strategy. Large, prospective studies are needed to fully explore the effect of post-vaccination fever on the developing immune response.

## Figures and Tables

**Table 1:** Baseline characteristics of study participants based on post-vaccination adverse event status. Data are presented as means with standard deviation or median with interquartile range as appropriate. Proportions are expressed both as numbers and percentages. A *p*-value less than 0.05 was considered statistically significant. BMI, body mass index; ACE-inhibitors, Angiotensin-converting enzyme-inhibitors; COVID-19, Coronavirus disease; NS, non-significant.

	Total Population (*N* = 383)	Asymptomatic Group (*N* = 214)	Symptomatic Group (*N* = 169)	*p*-Value
Age, (mean ± SD)	46.5 ± 12	47.6 ± 12	45.3 ± 12	NS
Female, (*N*, %)	303 (76.7)	159 (74.3)	139 (82.2)	NS
BMI, (mean ± SD)	27.6 ± 6	28.1 ± 7	26.9 ± 5	NS
Smoking, (*N*, %)	123 (34.7)	73 (37.2)	50 (31.8)	NS
Flu vaccination, (*N*, %)	67 (17.6)	37 (17.5)	30 (17.9)	NS
Hypertension, (*N*, %)	95 (26)	55 (28.2)	40 (23.7)	NS
Diabetes, (*N*, %)	22 (6)	14 (7.2)	8 (4.7)	NS
Hypothyreosis, (*N*, %)	25 (6.9)	15 (7.7)	10 (5.9)	NS
Autoimmune disease, (*N*, %)	20 (5.5)	10 (5.2)	10 (5.9)	NS
Allergy, (*N*, %)	96 (26.2)	36 (18.3)	60 (35.5)	<0.001
ACE inhibitors, (*N*, %)	63 (17.4)	34 (17.6)	29 (17.2)	NS
Beta blockers, (*N*, %)	60 (16.5)	30 (15.5)	30 (17.8)	NS
Calcium channel blocker, (*N*, %)	25 (6.9)	9 (4.7)	16 (9.5)	NS
Prior COVID-19 infection, (*N*, %)	85 (23.2)	47 (24)	38 (22.5)	NS

**Table 2:** Serial associations among level of S-IgG, adverse events after vaccination and demographic-clinical variables at seven time points (Day 12, Day 30, Day 60, Day 90, Day 120, Day 150, and Day 180 following second vaccine dose, respectively). § In this binary logistic regression model, serum S-IgG levels were converted to a binary dependent variable based on the median value of the sample (0: ≤median, 1: >median).

Variable	B	Odds Ratio	95% C.I.		*p*-Value
Day 12, value of S-IgG (AU/mL, median as the cutoff) §					
Fever, 2nd	−1.264	0.283	0.107	0.747	0.011
Day 30, value of S-IgG (AU/mL, median as the cutoff) §					
Fever, 2nd	−1.349	0.260	0.091	0.741	0.012
Day 60, value of S-IgG (AU/mL, median as the cutoff) §					
Smoking	0.651	1.917	1.157	3.176	0.012
Fever, 2nd	−1.372	0.254	0.086	0.748	0.013
Day 90, value of S-IgG (AU/mL, median as the cutoff) §					
Chills, 1st	−1.672	0.188	0.038	0.937	0.041
Fever, 2nd	−2.482	0.084	0.018	0.389	0.002
Day 120, value of S-IgG (AU/mL, median as the cutoff) §					
Age	−0.037	0.964	0.941	0.988	0.003
Smoking	0.780	2.181	1.227	3.878	0.008
Prior COVID+	−1.159	0.314	0.150	0.659	0.002
Fever, 2nd	−2.518	0.081	0.017	0.382	0.002
Day 150, value of S-IgG (AU/mL, median as the cutoff) §					
Prior COVID+	−0.781	0.458	0.216	0.972	0.042
Fever, 2nd	−2.414	0.089	0.019	0.413	0.002
Day 180, value of S-IgG (AU/mL, median as the cutoff) §					
Smoking	0.651	1.918	1.100	3.345	0.022
Fever, 2nd	−1.632	0.196	0.062	0.612	0.005

B, B coefficient; odds ratio, the exponentiation of the B coefficient EXP(B); 95%CI, 95% confident interval; S-IgG, anti-spike immunoglobulin; AU, arbitrary unit; COVID-19, confirmed corona virus disease-19.

## Data Availability

All relevant data are within the manuscript.

## Supplementary Materials

The following supporting information can be downloaded at: https://www.mdpi.com/article/10.3390/vaccines10030447/s1. Table S1: Frequency of adverse events after 1st and 2nd vaccination. Table S2: Correlation of S-IgG antibody levels with adverse reactions after 1st and 2nd dose of BNT162b2 vaccine manufactured by Pfizer/BioNTech, during the 6-month follow-up period. Table S3: Correlation of S-IgG antibody levels with demographic and clinical factors after 2nd dose of BNT162b2 vaccine manufactured by Pfizer/BioNTech, during the 6-month follow-up period. Table S4: Results of the binary logistic regression analysis examining associations between level of S-IgG, adverse events after vaccination and demographic-clinical variables at seven time points, namely, 12 and 30, 60, 90, 120, 150, and 180 days following second vaccine doses (designated Day 12, Day 30, Day 60, Day 90, Day 120, Day 150, and Day 180, respectively).

## References

[1] https://www.who.int/emergencies/diseases/novel-coronavirus-2019.

[2] Polack F.P., Thomas S.J., Kitchin N., Absalon J., Gurtman A., Lockhart S., Perez J.L., Pérez Marc G., Moreira E.D., Zerbini C. (2020). Safety and Efficacy of the BNT162b2 mRNA COVID-19 Vaccine. N. Engl. J. Med..

[3] Baden L.R., El Sahly H.M., Essink B., Kotloff K., Frey S., Novak R., Diemert D., Spector S.A., Rouphael N., Creech B. (2021). Efficacy and Safety of the mRNA-1273 SARS-CoV-2 Vaccine. N. Engl. J. Med..

[4] Yoda T., Katsuyama H. (2021). Willingness to Receive COVID-19 Vaccination in Japan. Vaccines.

[5] https://ourworldindata.org/COVID-vaccinations.

[6] Coggins S.A.A., Laing E.D., Olsen C.H., Goguet E., Moser M., Jackson-Thompson B.M., Samuels E.C., Pollett S.D., Tribble D.R., Davies J. (2022). Adverse Effects and Antibody Titers in Response to the BNT162b2 MRNA COVID-19 Vaccine in a Prospective Study of Healthcare Workers. Open Forum. Infect. Dis..

[7] Uwamino Y., Kurafuji T., Sato Y., Tomita Y., Shibata A., Tanabe A., Yatabe Y., Noguchi M., Arai T., Ohno A. (2022). Young age, female sex, and presence of systemic adverse reactions are associated with high post-vaccination antibody titer after two doses of BNT162b2 mRNA SARS-CoV-2 vaccination: An observational study of 646 Japanese healthcare workers and university staff. Vaccine.

[8] Moderbacher C., Ramirez S.I., Dan J.M., Grifoni A., Hastie K.M., Weiskopf D., Belanger S., Abbott R.K., Kim C., Choi J. (2020). Antigen-Specific Adaptive Immunity to SARS-CoV-2 in Acute COVID-19 and Associations with Age and Disease Severity. Cell.

[9] Tarbiah N., Todd I., Tighe P.J., Fairclough L.C. (2019). Cigarette smoking differentially affects immunoglobulin class levels in serum and saliva: An investigation and review. Basic Clin. Pharmacol. Toxicol..

[10] McMillan S.A., Douglas J.P., Archbold G.P., McCrum E.E., Evans A.E. (1997). Effect of low to moderate levels of smoking and alcohol consumption on serum immunoglobulin concentrations. J. Clin. Pathol..

[11] Qiu F., Liang C.L., Liu H., Zeng Y.Q., Hou S., Huang S., Lai X., Dai Z. (2017). Impacts of cigarette smoking on immune responsiveness: Up and down or upside down?. Oncotarget.

[12] Skok M.V., Grailhe R., Agenes F., Changeux J.P. (2007). The role of nicotinic receptors in B-lymphocyte development and activation. Life Sci..

[13] Fischinger S., Boudreau C.M., Butler A.L., Streeck H., Alter G. (2019). Sex differences in vaccine-induced humoral immunity. Semin. Immunopathol..

[14] Fathi A., Addo M.M., Dahlke C. (2021). Sex Differences in Immunity: Implications for the Development of Novel Vaccines against Emerging Pathogens. Front. Immunol..

[15] Naaber P., Tserel L., Kangro K., Sepp E., Jürjenson V., Adamson A., Haljasmägi L., Rumm A.P., Maruste R., Kärner J. (2021). Dynamics of antibody response to BNT162b2 vaccine after six months: A longitudinal prospective study. Lancet Reg. Health.

[16] Hanson D.F. (1997). Fever, temperature, and the immune response. Ann. N. Y. Acad. Sci..

[17] Evans S.S., Repasky E.A., Fisher D.T. (2015). Fever and the thermal regulation of immunity: The immune system feels the heat. Nat. Rev. Immunol..

[18] Müller L., Andrée M., Moskorz W., Drexler I., Walotka L., Grothmann R., Ptok J., Hillebrandt J., Ritchie A., Rabl D. (2021). Age-dependent Immune Response to the Biontech/Pfizer BNT162b2 Coronavirus Disease 2019 Vaccination. Clin. Infect. Dis..

[19] Andrews N.J., Walker W.T., Finn A., Heath P.T., Collinson A.C., Pollard A.J., Snape M.D., Faust S.N., Waight P.A., Hoschler K. (2011). Predictors of immune response and reactogenicity to AS03B-adjuvanted split virion and non-adjuvanted whole virion H1N1 (2009) pandemic influenza vaccines. Vaccine.

[20] Held J., Esse J., Tascilar K., Steininger P., Schober K., Irrgang P., Alsalameh R., Tenbusch M., Seggewies C., Bogdan C. (2021). Reactogenicity Correlates Only Weakly with Humoral Immunogenicity after COVID-19 Vaccination with BNT162b2 mRNA (Comirnaty^®^). Vaccines.

[21] Hwang Y.H., Song K.H., Choi Y., Go S., Choi S.J., Jung J., Kang C.K., Choe P.G., Kim N.J., Park W.B. (2021). Can reactogenicity predict immunogenicity after COVID-19 vaccination?. Korean J. Intern. Med..

[22] Otani J., Ohta R., Sano C. (2021). Association between Immunoglobulin G Levels and Adverse Effects Following Vaccination with the BNT162b2 Vaccine among Japanese Healthcare Workers. Vaccines.

[23] Prymula R., Siegrist C.A., Chlibek R., Zemlickova H., Vackova M., Smetana J., Lommel P., Kaliskova E., Borys D., Schuerman L. (2009). Effect of prophylactic paracetamol administration at time of vaccination on febrile reactions and antibody responses in children: Two open-label, randomised controlled trials. Lancet.

[24] Bancos S., Bernard M.P., Topham D.J., Phipps R.P. (2009). Ibuprofen and other widely used non-steroidal anti-inflammatory drugs inhibit antibody production in human cells. Cell Immunol..

[25] Paccani S.R., Boncristiano M., Ulivieri C., D’Elios M.M., Del Prete G., Baldari C.T. (2002). Nonsteroidal anti-inflammatory drugs suppress T-cell activation by inhibiting p38 MAPK induction. J. Biol. Chem..

[26] Ryan E.P., Pollock S.J., Murant T.I., Bernstein S.H., Felgar R.E., Phipps R.P. (2005). Activated human B lymphocytes express cyclooxygenase-2 and cyclooxygenase inhibitors attenuate antibody production. J. Immunol..

[27] Saleh E., Moody M.A., Walter E.B. (2016). Effect of antipyretic analgesics on immune responses to vaccination. Hum. Vaccines Immunother..

[28] Morales-Núñez J.J., Muñoz-Valle J.F., Meza-López C., Wang L.F., Machado Sulbarán A.C., Torres-Hernández P.C., Bedolla-Barajas M., De la O-Gómez B., Balcázar-Félix P., Hernández-Bello J. (2021). Neutralizing Antibodies Titers and Side Effects in Response to BNT162b2 Vaccine in Healthcare Workers with and without Prior SARS-CoV-2 Infection. Vaccines.

[29] Ali H., Alahmad B., Al-Shammari A.A., Alterki A., Hammad M., Cherian P., Alkhairi I., Sindhu S., Thanaraj T.A., Mohammad A. (2020). Previous COVID-19 Infection and Antibody Levels after Vaccination. Front. Public Health.

[30] Ebinger J.E., Fert-Bober J., Printsev I., Wu M., Sun N., Prostko J.C., Frias E.C., Stewart J.L., Van Eyk J.E., Braun J.G. (2021). Antibody responses to the BNT162b2 mRNA vaccine in individuals previously infected with SARS-CoV-2. Nat. Med..

[31] Xiao K., Yang H., Liu B., Pang X., Du J., Liu M., Liu Y., Jing X., Chen J., Deng S. (2021). Antibodies Can Last for More Than 1 Year After SARS-CoV-2 Infection: A Follow-Up Study From Survivors of COVID-19. Front. Med..

[32] Haas J.W., Bender F.L., Ballou S., Kelley J.M., Wilhelm M., Miller F.G., Rief W., Kaptchuk T.J. (2022). Frequency of Adverse Events in the Placebo Arms of COVID-19 Vaccine Trials: A Systematic Review and Meta-analysis. JAMA.

