# Supplementary material for: Fever after Vaccination against SARS-CoV-2 with mRNA-Based Vaccine Associated with Higher Antibody Levels during 6 Months Follow-Up
Source: Vaccines (Basel). 2022 Mar 14;10(3):447. doi: 10.3390/vaccines10030447 (PMC8950492; doi:10.3390/vaccines10030447)
Supplement: Supplementary file 1 [file vaccines-10-00447-s001.zip › Table S1.pdf]

	Number of adverse reactions after 1st dose (N=314)	Number of adverse reactions after 2nd dose (N=365)	Sum of adverse reactions after each dose (N=679)
Myalgia	77	112	189
Local pain	74	60	134
Fatigue	38	60	98
Fever	57	37	94
Headache	18	30	48
Chills	19	24	43
Arthralgia	8	17	25
Other	23	25	48

Table S1. Frequency of adverse reactions after 1st and 2nd vaccination. Only symptoms that occurred immediately after vaccination and for 7 days thereafter were considered vaccination adverse reactions.
