# Supplementary material for: Fever after Vaccination against SARS-CoV-2 with mRNA-Based Vaccine Associated with Higher Antibody Levels during 6 Months Follow-Up
Source: Vaccines (Basel). 2022 Mar 14;10(3):447. doi: 10.3390/vaccines10030447 (PMC8950492; doi:10.3390/vaccines10030447)
Supplement: Supplementary file 1 [file vaccines-10-00447-s001.zip › Table S2.pdf]

	Day 12 IgM	Day 12 IgG	Day 30 IgG	Day 60 IgG	Day 90 IgG	Day 120 IgG	Day 150 IgG	Day 180 IgG
After 1st dose								
Myalgia	-0.044	0.027	0.113*	0.092	0.120*	0.204**	0.153*	0.141*
Local pain	0.030	0.103	0.105	0.063	0.102	0.101	0.091	0.094
Fatigue	-0.061	0.050	0.027	0.004	0.041	0.063	0.058	0.097
Fever	-0.061	0.047	0.127*	0.091	0.131*	0.216**	0.212**	0.185**
Headache	0.028	0.077	0.113*	0.089	0.115*	0.081	0.080	0.080
Chills	-0.026	0.124*	0.167**	0.178**	0.185**	0.215**	0.195**	0.232**
Arthralgia	-0.074	-0.002	-0.021	0.017	-0.029	-0.007	-0.091	-0.059
After 2nd dose								
Myalgia	0.138**	0.181**	0.186**	0.129*	0.134*	0.110	0.149*	0.156*
Local pain	0.023	0.038	0.041	0.038	0.067	0.055	0.026	0.060
Fatigue	0.076	0.151*	0.130*	0.093	0.077	0.065	0.060	0.074
Fever	0.036	0.196**	0.246**	0.236**	0.296**	0.305**	0.362**	0.311**
Headache	0.073	0.129*	0.174**	0.163**	0.187**	0.155*	0.165*	0.180**
Chills	0.126*	0.217**	0.232**	0.236**	0.220**	0.201**	0.217**	0.184**
Arthralgia	-0.039	0.049	0.055	0.038	-0.033	-0.031	-0.011	-0.049

Table S2. Correlation of S-Ig antibody levels with adverse reactions after 1st and 2nd dose of BNT162b2 vaccine manufactured by Pfizer/BioNTech, during the 6-month follow-up period. Values are Spearman correlation coefficients. *$p<0.05$, **$p<0.01$
