# Supplementary material for: Fever after Vaccination against SARS-CoV-2 with mRNA-Based Vaccine Associated with Higher Antibody Levels during 6 Months Follow-Up
Source: Vaccines (Basel). 2022 Mar 14;10(3):447. doi: 10.3390/vaccines10030447 (PMC8950492; doi:10.3390/vaccines10030447)
Supplement: Supplementary file 1 [file vaccines-10-00447-s001.zip › Table S3.pdf]

	Day 12 IgG	Day 30 IgG	Day 60 IgG	Day 90 IgG	Day 120 IgG	Day 150 IgG	Day 180 IgG
Gender	0.088	0.050	0.041	0.044	0.002	0.055	0.040
Smoking	-0.107*	-0.134*	-0.177*	-0.142*	-0.164*	-0.091	-0.187**
BMI	-0.006	0.016	-0.024	-0.092	-0.057	-0.018	-0.001
Autoimmunity	-0.121*	-0.119*	-0.167**	-0.138*	-0.153*	-0.122	-0.054
Allergy	0.056	0.111*	0.118*	0.154**	0.106	0.088	0.080
ACE inhibitors	-0.126*	-0.117*	-0.112*	-0.105	-0.087	-0.047	-0.065
Contraceptives	0.121*	0.117*	0.101	0.096	0.114	0.128	0.120
Statins	-0.084	-0.096	-0.095	-0.121*	-0.121*	-0.153*	-0.181**
Hyperlipidaemia	-0.074	-0.085	-0.073	-0.092	-0.124*	-0.151*	-0.081

Table S3. Correlation of S-Ig antibody levels with demographic and clinical factors after 2nd dose of BNT162b2 vaccine manufactured by Pfizer/BioNTech, during the 6-month follow-up period. Values are Spearman correlation coefficients. *$p<0.05$, **$p<0.01$
