# Supplementary material for: Fever after Vaccination against SARS-CoV-2 with mRNA-Based Vaccine Associated with Higher Antibody Levels during 6 Months Follow-Up
Source: Vaccines (Basel). 2022 Mar 14;10(3):447. doi: 10.3390/vaccines10030447 (PMC8950492; doi:10.3390/vaccines10030447)
Supplement: Supplementary file 1 [file vaccines-10-00447-s001.zip › Table S4.pdf]

Table S4. Results of the binary logistic regression analysis examining associations between level of S-Ig, adverse events after vaccination and demographic-clinical variables at seven time points, namely, 12 and 30, 60, 90, 120, 150 and 180 days following second vaccine doses (designated Day 12, Day 30, Day 60, Day 90, Day 120, Day 150 and Day 180, respectively). § In these binary logistic regression models, serum S-Ig levels were converted to a binary dependent variable, based on the median value of the sample (0: ≤median, 1: >median).

A. Day 12, value of S-Ig (AU/mL, median as the cutoff) §

	B	S.E.	Wald	df	Sig.	Exp(B)	95% C.I.for EXP(B)	
							Lower	Upper
Gender	-0.078	0.264	0.087	1	0.768	0.925	0.551	1.553
Chills	-0.915	0.610	2.254	1	0.133	0.400	0.121	1.323
Myalgia, 2nd	-0.282	0.372	0.574	1	0.449	0.754	0.364	1.565
Headache, 2nd	-0.520	0.503	1.071	1	0.301	0.594	0.222	1.592
Age	-0.013	0.010	1.787	1	0.181	0.987	0.969	1.006
Prior COVID +	0.450	0.265	2.868	1	0.090	1.568	0.932	2.637
Fever, 2nd	-1.264	0.496	6.491	1	0.011	0.283	0.107	0.747

B. Day 30, value of S-Ig (AU/mL, median as the cutoff) §

	B	S.E.	Wald	df	Sig.	Exp(B)	95% C.I.for EXP(B)	
							Lower	Upper
Fever, 2nd	-1.349	0.535	6.349	1	0.012	0.260	0.091	0.741
Smoking	0.491	0.256	3.681	1	0.055	1.634	0.989	2.700
Headache, 2nd	-0.372	0.516	0.520	1	0.471	0.689	0.251	1.895
Myalgia, 2nd	-0.339	0.403	0.706	1	0.401	0.712	0.323	1.571
Chills, 2nd	-0.954	0.693	1.896	1	0.169	0.385	0.099	1.498
Age	-0.013	0.011	1.396	1	0.237	0.987	0.966	1.008
Prior COVID+	0.073	0.288	0.064	1	0.801	1.075	0.612	1.890
Gender	0.179	0.298	0.362	1	0.547	1.196	0.667	2.145

C. Day 60, value of S-Ig (AU/mL, median as the cutoff) §

	B	S.E.	Wald	df	Sig.	Exp(B)	95% C.I.for EXP(B)	
							Lower	Upper
Gender	0.269	0.297	0.820	1	0.365	1.309	0.731	2.345
Chills, 2nd	-0.268	0.664	0.162	1	0.687	0.765	0.208	2.812
Chills, 1st	-1.029	.618	2.777	1	0.096	0.357	0.106	1.199
Myalgia, 2nd	-0.067	0.399	0.028	1	0.868	0.936	0.428	2.047
Headache, 2nd	-0.475	0.528	0.808	1	0.369	0.622	0.221	1.752
Age	-0.021	0.011	3.747	1	0.053	0.979	0.958	1.000
Smoking	0.651	0.258	6.375	1	0.012	1.917	1.157	3.176
Prior COVID+	-0.191	0.302	0.399	1	0.527	0.826	0.457	1.493
Fever, 2nd	-1.372	0.551	6.188	1	0.013	0.254	0.086	0.748

D. Day 90, value of S-Ig (AU/mL, median as the cutoff) §

	B	S.E.	Wald	df	Sig.	Exp(B)	95% C.I.for EXP(B)	
							Lower	Upper
Gender	0.301	0.313	0.929	1	0.335	1.352	.732	2.494
Chills, 2nd	-0.397	0.758	0.274	1	0.601	0.673	0.152	2.971
Chills, 1st	-1.672	0.820	4.158	1	0.041	0.188	0.038	0.937
Headache, 2nd	-1.145	0.622	3.393	1	0.065	0.318	0.094	1.076
Age	-0.019	0.012	2.409	1	0.121	.981	0.958	1.005
ACE-inhibitor	0.552	0.371	2.212	1	0.137	1.736	0.839	3.592
Smoking	0.510	0.273	3.483	1	0.062	1.666	0.975	2.846
Prior COVID+	-0.352	0.335	1.109	1	0.292	0.703	0.365	1.354
Fever, 2nd	-2.482	0.784	10.020	1	0.002	0.084	0.018	0.389

E. Day 120, value of S-Ig (AU/mL, median as the cutoff) §

	B	S.E.	Wald	df	Sig.	Exp(B)	95% C.I.for EXP(B)	
							Lower	Upper
Gender	0.234	0.348	0.451	1	0.502	1.263	0.639	2.499
Chills, 2nd	-0.656	0.744	0.776	1	0.378	0.519	0.121	2.232
Age	-0.037	0.013	8.557	1	0.003	0.964	0.941	0.988
Smoking	0.780	0.294	7.054	1	0.008	2.181	1.227	3.878
Prior COVID+	-1.159	0.378	9.380	1	0.002	0.314	0.150	0.659
Fever, 2nd	-2.518	0.795	10.046	1	0.002	0.081	0.017	0.382

F. Day 150, value of S-Ig (AU/mL, median as the cutoff) §

	B	S.E.	Wald	df	Sig.	Exp(B)	95% C.I.for EXP(B)	
							Lower	Upper
Gender	-0.314	0.392	0.642	1	0.423	0.730	0.339	1.575
Chills, 2nd	-0.403	0.792	0.259	1	0.611	0.669	0.142	3.155
Chills, 1st	-0.835	0.739	1.274	1	0.259	0.434	0.102	1.849
Age	-0.016	0.013	1.366	1	0.243	0.984	0.959	1.011
Headache, 2nd	-0.872	0.643	1.839	1	0.175	0.418	0.119	1.474
Smoking	0.318	0.321	0.985	1	0.321	10.375	0.733	2.577
Prior COVID+	-0.781	0.384	4.133	1	0.042	0.458	0.216	0.972
Fever, 2nd	-2.414	0.781	9.554	1	0.002	0.089	0.019	0.413

G. Day 180, value of S-Ig (AU/mL, median as the cutoff) §

	B	S.E.	Wald	df	Sig.	Exp(B)	95% C.I.for EXP(B)	
							Lower	Upper
Gender	0.046	0.348	0.018	1	0.894	1.048	0.530	2.070
Chills, 1st	-1.552	0.809	3.676	1	0.055	0.212	0.043	1.035
Age	-0.015	0.012	1.424	1	0.233	0.985	0.962	1.009
Myalgia, 2nd	-0.298	0.411	0.527	1	0.468	0.742	0.332	1.660
Smoking	0.651	0.284	5.273	1	0.022	1.918	1.100	3.345
Priod COVID+	-0.683	0.355	3.700	1	0.054	0.505	0.252	1.013
Fever, 2nd	-1.632	0.582	7.852	1	0.005	0.196	0.062	0.612
